# Que(e) rying undergraduate medical curricula: a cross-sectional online survey of lesbian, gay, bisexual, transgender, and queer content inclusion in UK undergraduate medical education

**DOI:** 10.1186/s12909-021-02532-y

**Published:** 2021-02-12

**Authors:** Nicholas Tollemache, Duncan Shrewsbury, Carrie Llewellyn

**Affiliations:** Department of Primary Care and Public Health, Brighton and Sussex Medical School, Falmer, Sussex, Brighton, BN19PU UK

**Keywords:** Medical education, Inclusion, LGBTQ+ / lesbian, gay, bisexual, trans, queer, Health inequalities, Sexual orientation, Healthcare

## Abstract

**Background:**

Lesbian, Gay, Bisexual, and Transgender (LGBT) individuals are more likely to have negative healthcare experiences and worse health outcomes when compared with their heterosexual and cisgender counterparts. A key recommendation of the 2018 Stonewall-commissioned “LGBT in Britain” report was that the curricula, standards, and training provided by medical schools should be reviewed in order to encompass mandatory teaching about LGBT health inequalities and discrimination, LGBT-inclusive care and the use of appropriate language. The aim of our study was to conduct an in-depth national review of the content of LGBT teaching within the curricula of UK Medical Schools.

**Methods:**

Course leads at all 37 UK Medical Schools with students currently enrolled in a primary undergraduate medical training course were asked between December 2019–March 2020 to complete a cross-sectional online survey comprised of 30 questions; divided into three sections relating to the current LGBT teaching (Part 1), any planned or future LGBT teaching (Part 2), and the opinions of the survey respondent about the coverage of LGBT topics (Part 3) at their institution. Responses were analysed using descriptive statistics.

**Results:**

Questionnaires were received from 19/37 institutions (response rate: 51%). The median estimated number of hours of LGBT-teaching across the entire undergraduate course was 11.0 (IQR: 12.25). Teaching on LGBT mental health, gender identity, sexual orientation, awareness of LGBT-health inequalities, and LGBT discrimination in healthcare were reported by almost all respondents, whilst maternity and childbirth, chronic disease and LGBT adolescent health were least represented within the curriculum. Almost all (18 medical schools; 95%) responding institutions were considering implementing new LGBT teaching within the next three academic years. A lack of space within the curriculum is a universally reported barrier to the implementation of LGBT teaching. Only 5 (26%) survey respondents consider their institution’s current coverage of LGBT topics to be “Good” or “Very good”.

**Conclusion:**

Our study demonstrates a significant variation in the amount and breadth of content within the undergraduate curricula of UK medical schools. Recommendations for increasing the quantity and quality of LGBT content are provided, based upon areas of good practice.

**Supplementary Information:**

The online version contains supplementary material available at 10.1186/s12909-021-02532-y.

## Background

In the United Kingdom (UK), at least 2.4% of the population identifies as Lesbian, Gay, or Bisexual [1] and it is estimated that 0.2–0.6% of the population identifies as transgender or non-binary [2]. It is well documented that Lesbian, Gay, Bisexual, and Transgender (LGBT) individuals have unique healthcare needs and are more likely to have negative healthcare experiences and worse health outcomes when compared with their heterosexual and cisgender counterparts [3]. This begins with access to appropriate healthcare services; LGBT individuals are more likely to delay or avoid seeking treatment and are reluctant to disclose their sexual orientation to healthcare professionals [4, 5]. A systematic review of sexual orientation disclosure identified a number of facilitators and barriers covering the moment of disclosure, the patient-perceived outcome of the disclosure, the healthcare professional(s) involved, and environmental factors [6].

The specific needs of the LGBT population encompasses both physical and mental health. In using the abbreviation ‘LGBT’ and not other more inclusive terms (such as LGBTQ+ or LGBTQIA) to refer to diverse communities, we acknowledge the risks of under-representation and implied homogeny inherent with labelling. Terminology in this field is dynamic and consensus is not uniformly agreed. Our choice of terminology reflects extant literature, with the conscious intention to be inclusive of and sensitive to the needs of people who identify as queer, non-binary, asexual and other gender and sexual diversities that may experience marginalisation or inequity. The intention of this work is to shine a light on this broad and dynamic field, through access negotiated by terminology that has gained currency and shared understanding. The experiences and needs of, and risks to the individual communities within this broader group are each unique and complex, although often discussed in combination as ‘other’ from a cis-gendered heterosexual norm. LGBT individuals are at increased risk of certain cancers, have higher rates of chronic disease, Human Immunodeficiency Virus (HIV) infection, and sexually-transmitted infections (STIs) [7, 8]. With respect to mental health, LGBT people are at higher risk of developing anxiety and depression, and are more likely to self-harm or attempt suicide [9]. People in LGBT communities are also more likely to engage in high-risk health behaviours, such as tobacco usage, excessive alcohol consumption, and substance misuse [10]. Domestic violence is also more prevalent in the LGBT population, either as a result of intimate partner violence (IPV) or violence against children, adolescents, and young people who live in the family home [11]. The UK has an aging population, and older LGBT people are likely to face additional challenges when compared to heterosexual and cisgender individuals [12]. A survey conducted in the UK as recently as 2018 identified that LGBT people were likely to face discriminatory attitudes, inappropriate curiosity with respect to their gender and sexuality, and a lack of awareness of their differing needs, from healthcare professionals [13]. A key recommendation of the report was that the “curricula, standards, and training” of medical schools should be reviewed in order to encompass mandatory teaching on the use of appropriate language, LGBT health inequalities and discrimination, and LGBT-inclusive care.

Medical students are aware of their own limitations in caring for LGBT patients; in 2016, a survey of medical students across all academic years at The University of Oxford found that 85% of the 166 respondents reported a lack of teaching on specific LGBT health needs within their course, with particular deficits in their self-reported ability to care for LGBT patients in the clinical setting and their knowledge of LGBT-specific terminology [14]. Educational programmes about the specific health needs of LGBT individuals can improve the knowledge and attitudes of healthcare students and professionals [15]. Although LGBT needs are not articulated explicitly, the teaching of inclusive approaches to patients and colleagues, sensitivity to factors that may influence marginalisation and vulnerability, and a respect for diversity are mandated in the regulation of undergraduate medical curriculum [16].

Although research has been carried out into the LGBT curricula of US and Canadian medical training institutions [17], there have been no reports on the overall state of LGBT teaching in the undergraduate curricula of UK medical schools. Despite this, a few institutions have been proactive in documenting and disseminating their efforts in this area. In 2016, University College London (UCL), and Bristol medical schools have implemented sessions within their core teaching, to raise awareness of LGBT health inequalities. Both initiatives were evaluated positively by students, resulting in improvements in self-rated awareness of issues affecting, and confidence in meeting the needs of patients from LGBT communities [18–20]. In addition, there is evidence of early and developmental work at other medical schools, such as Edinburgh and Manchester, with the intention of integrating materials into core undergraduate curricula [21, 22].

Aside from these documented examples, the current and proposed status of LGBT-specific teaching within the curricula of other UK medical schools is unknown. Recent work suggests concern among medical students that knowledge and skills are not adequately adept to meet the needs of LGBT communities [23]. Therefore, it would behove socially responsible medical education to develop a clear understanding of areas of educational deficit alongside the dissemination of specific examples of good practice and practical measures that can be taken in the design and implementation of LGBT teaching within medical curricula.

### Aims

Therefore, the primary aim of our study was to conduct a national review of the content of LGBT teaching within the curricula of UK Medical Schools via a cross-sectional online survey; we intended to answer the following four research questions:

1. What is the current practice of UK medical schools in providing LGBT teaching as part of the undergraduate medical curriculum?
2. What are the intentions of UK medical schools’ curriculum development teams towards the creation of new LGBT teaching programmes?
3. What are the perceived and / or experienced barriers to designing and implementing LGBT teaching programmes into the undergraduate medical curriculum?
4. What are the opinions of survey respondents about the current LGBT teaching within their institution?

## Methods

### Design

The study design was a cross-sectional online survey.

### Sample and eligibility

There were 41 Medical Schools in the UK that offer a primary medical qualification (BMBS, MBChB, MBBS, MB BChir); four of these were excluded from our study as two had not currently enrolled any students at the time of the survey and two only offered graduate-entry degrees [24]. Although three Medical Schools have only delivered their curriculum across the 1st and 2nd years of the course, these were included within the eligibility criteria leading to a potential maximum sample of 37.

### Procedure

The survey was distributed via email in December 2019 to one undergraduate course lead at each of the eligible 37 Medical Schools using publicly available information from the Medical Schools Council [24] and individual institutional websites; follow-up contact was made with non-respondents by email and telephone. If it transpired that the available information was incorrect, then the administrative staff at the eligible Medical School were contacted for the updated details of the undergraduate course lead. It was made clear in all recruitment materials that the survey should be forwarded on internally to the faculty member with the greatest knowledge about LGBT-teaching within an institution. Participants were given until March 2020 to complete the survey, at which point the online survey link automatically deactivated.

Questionnaire responses were collected via Qualtrics XM (SAP SE, Walldorf, Germany) using an institutional licence (registered to the University of Sussex) and branded domain name (https://universityofsussex.eu.qualtrics.com/) with 256-bit SSL (Secure Socket Layer) encryption, in compliance with European Union General Data Protection Regulation 2016/679 (GDPR) and the University of Sussex’s Research Data Management Policy.

Informed consent was obtained from all individuals prior to completing the survey using an online form; this included a link to the participant information sheet and the contact details of all researchers, should the participant have further questions. To encourage candour and maintain confidentiality, participants were informed that individual institutions would not be identifiable in any resultant publications and that all data would be stored in an anonymised format upon submission. The survey platform allowed the initial respondent to complete the questionnaire and forward a link on to one other individual at their institution, who could review their responses to Parts 1 and 2 of the survey; secondary participants were prevented from viewing the Part 3 responses of the primary respondent and could submit their own responses for this part of the survey. Informed consent for the secondary participant was obtained using the same method as for the first participant.

### Questionnaire design

The questionnaire (Appendix 1) was comprised of thirty questions across three sections covering the current institutional practice regarding LGBT teaching (Part 1), the proposed or future institutional practice regarding LGBT teaching (Part 2), and the opinions of the survey respondent about current LGBT teaching within their institution (Part 3). Seven questions (Q3, Q4, Q7, Q8, Q14, Q21, and Q28) were adapted, with permission, from a similar study carried out across the United States and Canada by Obedin-Maliver et al. [17]; adaptions to these questions consisted of minor changes to the wording of the stems, and were carried out to account for the differences in curricula between UK and international medical schools and to better reflect our study’s research objectives.

With respect to our four research questions, Q1–18 and Q21 corresponded to research question 1: What is the current practice of UK medical schools in providing LGBT teaching as part of the undergraduate medical curriculum?; Q22 and Q23 corresponded to research question 2: What are the intentions of UK medical schools’ curriculum development teams towards the creation of new LGBT teaching programmes?; Q19, Q20, and Q24–26 corresponded to research question 3: What are the perceived and / or experienced barriers to designing and implementing LGBT teaching programmes into the undergraduate medical curriculum?; and Q28–30 corresponded to research question 4: What are the opinions of survey respondents about the current LGBT teaching within their institution?

The questionnaire used four main responses: multiple-choice questions, free-text questions, Likert scales, and matrix tables. All multiple-choice questions where more than one option could be selected, included an “other” choice which allowed respondents to input additional option(s) as free text; certain other multiple-choice questions enabled additional details to be provided in free text when the respondent answered “Yes”. Q9 was an open-ended question that allowed participants to provide detailed information about all current LGBT-teaching programmes at their institution. Q20 permitted respondents to describe, using free text, how the barriers to implementing the current LGBT-teaching were overcome.

The four questions (Q4, Q8, Q23, and Q30) that used a matrix table contained a fixed list of content areas pertinent to LGBT health inequalities and healthcare experiences (Table 1); these were adapted from Obedin-Maliver et al., who devised the original list from a MEDLINE search of appropriate terms [17]. The final topics were not meant to be exhaustive, but representative of potentially critical features of LGBT experiences that affect health and to which students may be exposed. The final topic list was endorsed by a panel of LGBT health and community health experts for accuracy, timeliness, and current priorities regarding health issues affecting LGBT communities. The originally drafted questionnaire was piloted with 13 deans of medical education by Obedin-Maliver et al. We did not conduct further substantiation outside of the incorporation of content by experts in the field [17]. Respondents were also able to add up to three additional content areas per question that they felt were not already represented by the aforementioned list. As part of the electronic survey formatting, respondents were required to answer all multiple-choice questions (Yes / No / Don’t Know), as well as all questions in Part 3 of the study (relating to their opinions about LGBT teaching); these questions were mandatory in order to maximise the degree of information captured in our questionnaire. The job title of the study participant was also collected and used for analysis; this was held separately to prevent identification of study responses. The questionnaire was reviewed and piloted by the whole project team, and subsequently refined before being finalised and distributed.

**Table 1:** Fixed list of content areas pertinent to LGBT health inequalities and healthcare experiences that appear in the four questioning containing matrix tables in the questionnaire (Qs 4, 8, 23, 30)

Maternity and childbirth in LGBT people.	
Chronic Disease in LGBT populations.	
LGBT Adolescent Health.	
Understanding of LGBT Families.	
Preventative health and cancer screening in LGBT people.	
Alcohol, tobacco, and illicit drug use in LGBT people.	
Sexually transmitted infections (not HIV) in LGBT people.	
Transitioning and Sex Reassignment Surgery.	
HIV in LGBT people.	
Communication skills with LGBT people.	
LGBT discrimination in healthcare.	
Awareness of LGBT-specific health inequalities.	
Sexual orientation.	
Gender Identity.	

### Data analysis

The questionnaire responses were coded by the researchers; in cases where more than one response was received from a single institution, the more positive and / or complete responses were combined to create a single institutional response. Descriptive analysis was carried out in Microsoft Excel 2019 (Microsoft Corp, Redmond, Washington, US) using percentages and simple statistical tests (e.g. mean, median, range). Where possible, results were presented graphically using pie charts and column charts. Responses to open-ended questions were organised into themes and summarised in a table.

### Ethical approval

Ethical approval was sought and obtained from the Brighton and Sussex Medical School (BSMS) Research, Governance and Ethics Committee (RGEC) on 26th November 2019 (ER/BSMS2913/1).

## Results

**Fig. 1:**
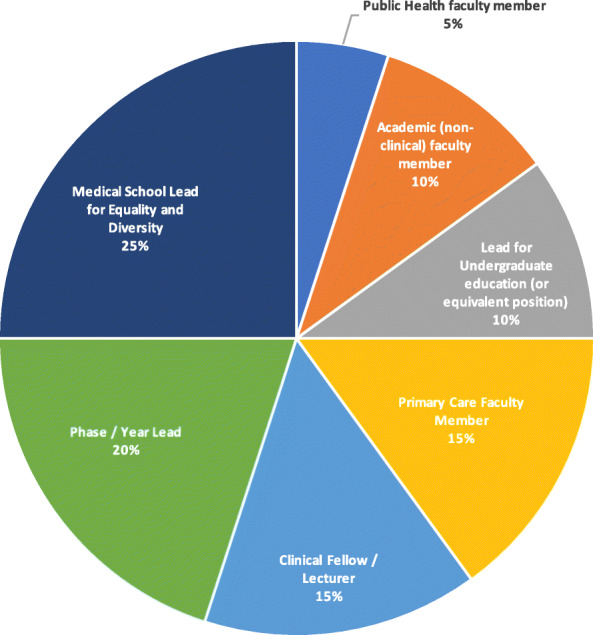
: Summary of survey respondent job roles

The overall response rate for our study was 51% (19/37); 18 medical schools (out of 37 eligible) submitted at least one completed questionnaire response and 1 institution asked for the response of another institution to also be applied to them (as they were following the same curriculum, with no plans to deviate from this blueprint). One medical school declined to participate. The remainder [17] of the eligible institutions did not respond. One questionnaire was completed by 16 institutions and 2 institutions completed two questionnaires. Three institutions were medical schools that had not yet delivered their curriculum beyond Year 2 and, therefore, were unable to answer questions related to the curriculum content in Years 3 to 5 of their course. A summary of survey respondents’ job roles is provided in Fig. 1.

### Current practice regarding LGBT teaching

The median reported number of hours of LGBT-teaching across the whole undergraduate course was 11.0 (IQR: 12.25); this ranged from a minimum of 3 h to a maximum of 55 h.

### Coverage of LGBT content areas

**Fig. 2:**
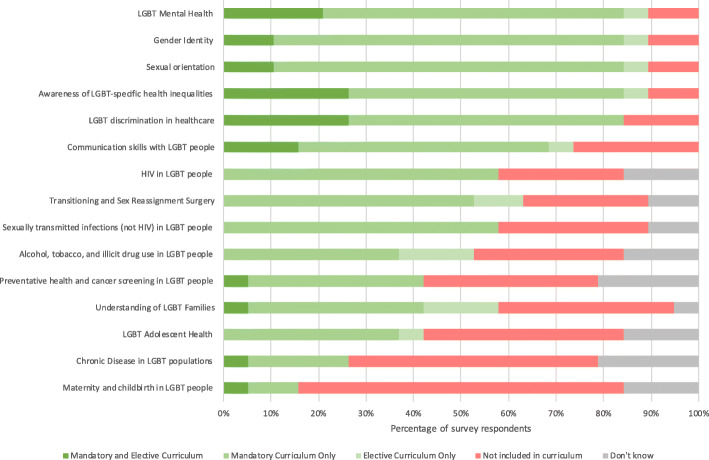
: Content areas included within the curriculum across all years of the undergraduate medical course

Across all years of the undergraduate medical course, the content areas covered least within the curriculum were “Maternity and childbirth in LGBT people”, “Chronic disease in LGBT populations”, and “LGBT Adolescent Health”; a summary of current content area inclusion is provided in Fig. 2.

### Teaching methods

**Fig. 3:**
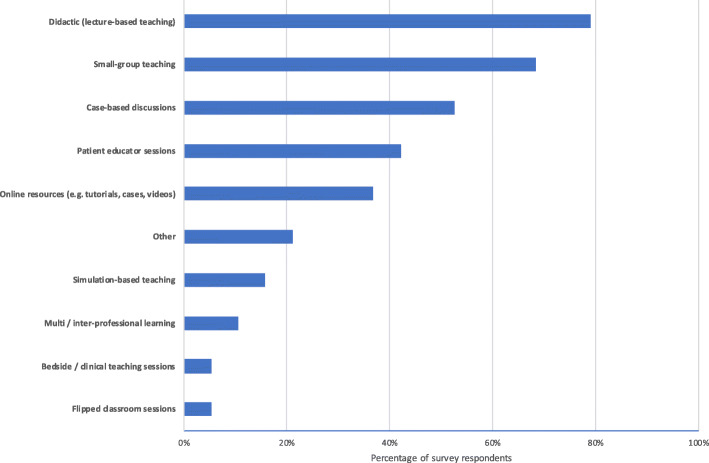
Summary of LGBT teaching methods

The majority of institutions used didactic (lecture-based) and small-group teaching to deliver LGBT-content to their students, whilst only a small number used simulation or bedside clinical teaching. A summary of LGBT-teaching methods is provided in Fig. 3.

### Examples of LGBT teaching

A summary of the free-text examples (Table 2) of specific LGBT teaching programmes, identified two key themes; firstly, the use of individuals (students, faculty members, and expert patients) who identify as LGBT as teachers or facilitators, and secondly, the use of charities and / or external organisations to deliver specific sessions or produce LGBT content.

**Table 2:** Summary of free-text quotations grouped by theme

LGBT Individuals as teachers or facilitators	Use of external organisations to deliver LGBT sessions or produce content	Other
*“Case based discussions of scenarios involving LGBT health workers with online resources to explore and inform discussion. Y3 Transgender awareness workshop run by an academic who is transgender”* Medical School 1 (**MS1**)	*“Awareness workshop run with [local county] LGBT, a local charity and advocacy organisation”* **MS1**	Informal approach to increased student exposure to LGBT topics: *“One format also tried was small group lunches with a volunteer in the room informally discussing their lived experiences both in and outside healthcare (*e.g. *how it feels to come out / question your sexuality or gender identity / family issues / relationships / microaggressions etc).”* **MS12**
“In year 1, the lecture on LGBT health is delivered by undergraduate medical students with experience of LGBT issues” **MS5**	“[we] utilise RCGP [the Royal College of General Practitioners] LGBT+ online modules” **MS9**	*“It is also important to keep track of where people are doing LGBTQ+ inclusive teaching so this can be reviewed - well-meaning educators including content which can sometimes be unintentionally problematic (playing into stereotypes or the educator not having enough knowledge,* e.g. *around trans people’s experience)”* **MS4**
“We have an interactive patient lecture” **MS9**	“A supporting lecture and Q&A session were provided by [local county trans organisation], our local Trans support charity” **MS14**	*“This initiative is … being maintained by a very small number of trained and interested academics and it is deemed above and beyond our role descriptions. This is however changing and we are starting to see review and change in the institution.”* **MS7**
“Year 5: 3 h session (lectures and small groups). Led by LGBT+ students and alumni with oversight and input from faculty (communications skills and public health) … a panel of LGBT+ volunteers [were] part of the session …” **MS12**	“… some is delivered in conjunction with LGBT organisations” **MS19**	

### Feedback and assessment

Feedback on LGBT teaching was collected at 11 institutions (58%). Students were formally assessed (either formatively or summatively) against the learning objectives from their LGBT teaching at 9 medical schools (47%); at these institutions, the most commonly used methods of assessment were: objective structured clinical examinations (OSCEs) (56%), coursework (44%), short answer questions (44%), multiple choice questions (44%), and as part of an (e) Portfolio (33%). One institution delivered content as part of a professional development module, of which non-engagement was considered a professionalism issue.

### Faculty training, resources, and content review

Faculty development for educators about LGBT-inclusive teaching was offered at ten medical schools (53%) with most respondents stating that this was provided by the parent university’s inclusion and diversity (or equivalent) department. Written resources were provided to educators at seven institutions (37%), although only one of these contained specific guidance pertaining to the creation of LGBT-inclusive learning materials. Although 11 institutions (58%) require educators to submit materials in advance in teaching sessions, only 1 reviewed this content for LGBT-inclusivity; this was highlighted as an area of importance in one free-text response (Table 2).

### Years 1 and 2

In the first two years of the course, 16 institutions (84%) taught LGBT-related content, of which the majority was integrated throughout the curriculum (69%) and delivered via didactic (81%) or small-group (63%) teaching.

### Years 3 to 5

In the final three years of the course, 13 institutions (81%) taught LGBT-related content. In addition to the content areas previously stated, only three institutions also provided mandatory or elective teaching on the handling of LGBT matters within the workplace, such as “how to escalate inappropriate remarks and deal with homophobic / transphobic colleagues”, “working with / being an ally [to] LGBT+ colleagues”, and “sexual violence in the workplace.”

### Proposed and / or future practice regarding LGBT teaching

**Fig. 4:**
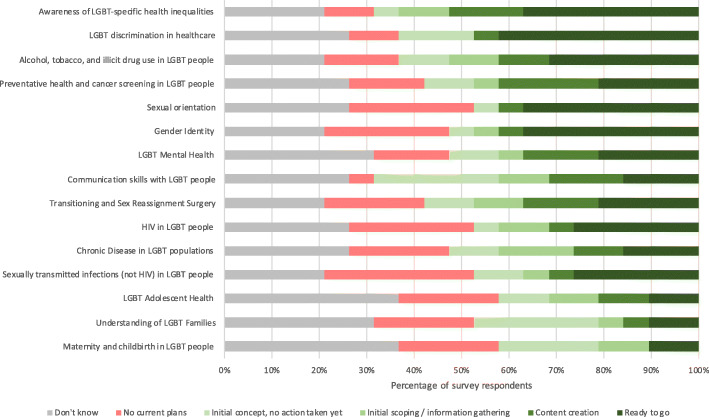
Planned LGBT teaching by content area

Almost all institutions (95%) were considering implementing new LGBT teaching within the next three academic years; the most commonly cited areas for future LGBT teaching were “Awareness of LGBT-specific health inequalities”, “LGBT discrimination in healthcare”, and “Alcohol, tobacco, and illicit drug use in LGBT people”; a summary of planned LGBT teaching is provided in Fig. 4.

### Barriers to LGBT teaching

The most frequently encountered barrier when implementing the current LGBT teaching programmes was a lack of space within the curriculum or time constraints (84%); this was overcome by delivering the content through e-learning or by integrating the teaching into pre-existing sessions (e.g. rewriting problem-based learning cases), themes, or modules (e.g. communication skills, public health).

Where there was a lack of experience within the faculty, or insufficient knowledge about the process of implementing LGBT teaching, a number of strategies were adopted; these included the use of LGBT charities to create and deliver content; collaboration with other universities, through organisations such as Diversity in Medicine and Health (DIMAH); utilisation of the personal and / or professional expertise of affiliated individuals, such as LGBT faculty members and local clinicians; the reorganisation of internal school structures to create a dedicated subject lead; and the recruitment of new staff with a special interest in LGBT teaching.

The commitment of specific, key faculty members was repeatedly cited as a positive force in overcoming the barriers associated with implementing new LGBT teaching; this was achieved by these individuals undertaking work outside of their formal job role and, by evidencing the impact of teaching, advocating for increased time, space, and inclusion of these topics in the curriculum. One respondent (MS7) noted that these actions may also act as a catalyst for both cultural and curricula change (Table 2).

A lack of the perceived relevance of LGBT teaching to students was not reported, however, three respondents experienced a lack of perceived importance institutionally and it was suggested that the scarcity of space within the curriculum could be a reason for this. A formal, school-wide curriculum review was mentioned by two respondents as an opportunity to consider the current coverage of LGBT topics and it appears that this was successful in at least one institution*.*

When planning future teaching sessions, a lack of space (teaching time) within the curriculum or time constraints was, again, the most cited barrier (89%), and at three institutions (18%) this prevented proposed teaching from proceeding.

### Opinions on current institutional LGBT teaching

**Fig. 5:**
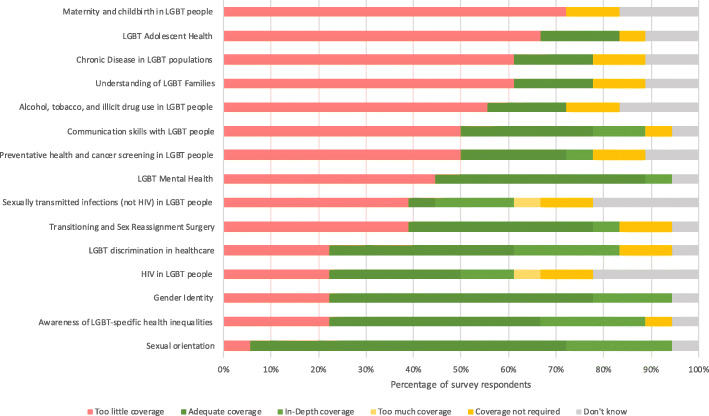
Survey respondents’ opinions on the current institutional coverage of LGBT content areas

Only 26% of survey respondents thought that the overall coverage of LGBT topics at their institution was either “good” or “very good”, whilst half (47%) believed that their institution “could do better”. Almost all participants (95%) considered that LGBT inequalities were at least “somewhat covered” by existing wider university inclusivity guidance and / or policies. A summary of survey respondents’ opinions about how well each content area is currently covered by their institution is provided in Fig. 5.

## Discussion

Our study is the first of its’ kind to explore the current state of LGBT teaching within the undergraduate curricula of UK medical schools; previous studies in this field have only investigated the knowledge and attitudes of UK medical students, or the effect of specific teaching interventions, rather than the quantity and content of LGBT teaching within the curriculum as a whole to provide a national picture [14, 19, 20].

### Principal findings

Our results suggest that teaching on LGBT-specific matters features, to some extent, in the curricula of almost all medical schools within our study, however, there is significant variation in the amount of time dedicated to the subject. We report a median total duration of 11 h of LGBT teaching across the whole undergraduate course; a similar study in the US and Canada from 2011 reported a lower median duration of 5 overall hours of LGBT teaching, although making a direct comparison is challenging due to the differences in curriculum, culture, age of the study, and the inclusion criteria used [17].

Regarding the content of current teaching, LGBT mental health, gender identity, sexual orientation, awareness of LGBT-health inequalities, and LGBT discrimination in healthcare, are almost universally covered in the mandatory curriculum of those medical schools surveyed. In contrast, maternity and childbirth in LGBT people, chronic disease in LGBT populations, LGBT adolescent health, and the understanding of LGBT families were noted as being poorly covered within the curriculum across the majority of institutions. The representation of these content areas is reflective of the literature [14, 18–20]. Similar content deficits have also been noted in South Africa, the US, and Canada [17, 25]. A critical view on policy around this area might suggest that an insufficiently explicit representation of LGBT needs within regulatory documentation is reflective of, if not contributory to, a corresponding insufficient recognition and prioritisation of this subject area [16].

In the increasingly clinical years of the course (years 3–5), the handling of LGBT issues within the workplace (e.g. inappropriate LGBT-associated remarks, homophobia, transphobia, and sexual violence) was highlighted as an additional topic that features in some curricula; this is a positive finding and echoes a 2015 Stonewall survey which reported that a quarter of health and social care staff members in patient-facing roles have heard negative or discriminatory remarks about LGBT people made by their colleagues in the prior five years, and 16% felt they were lacking in confidence to be able to challenge them [26].

Encouragingly, our findings show a strong inclination amongst medical schools to implement additional LGBT teaching, although this is limited to certain content areas, many of which are already represented to some extent within the curriculum, and there is a high degree of variation in how far through the process the surveyed institutions have progressed. Should this teaching be realised, it would likely result in positive outcomes, as medical students with a greater degree of exposure to LGBT patients have been shown to exhibit more positive attitudes and increased knowledge levels [27].

Our study also highlighted a number of barriers when planning or implementing LGBT teaching, with the most commonly cited being a lack of teaching time within the curriculum; this may be representative of a general unwillingness to instigate or promote a change in practice, although evidence suggests that this issue is not unique to this area [28]. To this end, medical schools and their faculty members have been creative in developing solutions to ensure that LGBT teaching occurs as part of the course; these include the incorporation of additional LGBT content into pre-existing teaching sessions or through curriculum reviews; evidencing the impact of LGBT teaching; internal or external collaborative effects; and through the undertaking of work outside of usual job roles.

Almost all schools surveyed did not provide educators with any written advice about the creation of LGBT-inclusive content and had no formal processes in place for the review or audit of teaching materials or sessions; whilst evaluating the teaching of distinct LGBT content is important, it should not be considered separate from the development of an inclusive curriculum, where LGBT patients feature in a range of educational scenarios and inclusive language is used throughout all teaching materials, as opposed to being siloed within specific modules, such as sexual health and endocrinology [29]. Overall, our study participants expressed a dissatisfaction with the coverage of LGBT topics at their own institutions, which is reflective of previous international research in this field [17].

### Strengths and limitations

Our study has a number of strengths. Firstly, the data collection was performed across a limited time period of four months in the middle of the academic year, which enabled us to capture a relatively static point in curriculum, where the addition or removal of content is less likely to have occurred; this increases the reliability of the data collected that relates to current teaching practices. Secondly, the questionnaire assessed each research question using multiple response types; this included the use of free-text questions, which enabled participants to provide a high level of detail about the current teaching programmes and their experiences implementing them. Thirdly, our survey was designed to be filled in by the person with the greatest knowledge of LGBT teaching in the curriculum and institutions were encouraged to identify and nominate this individual to complete the questionnaire on their behalf; this permitted the maximum degree of information to be obtained and reduced the degree of best guess or “don’t know” responses.

Despite these strengths, our study has a number of potential limitations. Firstly, although the overall response rate was acceptable), the data obtained can only be used to report directly on the 51% of medical schools offering an undergraduate qualification. A number of unifying themes were identified, but it would not be appropriate to generalise these results for the remainder of medical schools in the UK. Secondly, despite the fact that it was made clear, in advance, to participants that the results of the study would be anonymised, it is likely that there is a degree of social desirability bias within our study as a result of the person completing the questionnaire aiming to satisfy either personal or institutional goals and objectives. Our study was not designed to capture the depth of topic coverage within the curriculum to any degree greater than whether an area was present in the mandatory curriculum, elective curriculum, or both. Finally, in using the abbreviation ‘LGBT’ and not other terms such as LGBTQ+ or LGBTQIA to refer to diverse communities, we acknowledge the risks of under-representation and implied homogeny inherent with labelling. Future work should seek to understand the inclusion of content with regard to often marginalised voices such as those who identify as intersex, queer or asexual.

### Study implications and recommendations

Our study provides a national baseline that can be used by individual medical schools to comparatively evaluate the LGBT content of their curriculum. Organisations, such as the UK’s General Medical Council (GMC), that are responsible for defining and monitoring the outcomes for medical school graduates, may also use this information to inform the development of guidance for institutions on the inclusion of LGBT-specific learning outcomes [16]. Indeed, this work suggests a need for organisations with such responsibility to explicitly articulate and prioritise expectations of inclusive education in undergraduate curricula in regards to LGBT health needs. From a public and patient perspective, an accurate and transparent report on what is currently being taught to future clinicians regarding LGBT health may elicit feedback on under-represented issues important to the LGBT community and subsequent lobbying through national organisations for their inclusion in the curriculum.

Based upon the results of our study, a summary of suggested actions that may improve the institutional coverage of LGBT-related content is provided in Table 3.

**Table 3:** Summary of suggested actions to improve institutional LGBT-related coverage

Recommendation	Details
Creation of a dedicated lead role for LGBT topics within the curriculum	This can be fulfilled by: • An existing faculty member with an interest in LGBT teaching; or • The hire of an external individual with prior experience in delivering an LGBT-inclusive curriculum
External collaboration	This can be fulfilled by: • Collaboration with local or national LGBT charities on the development and delivery of teaching material that reflect both current and important LGBT health issues • Collaboration with other institutions (universities and medical schools) in the sharing of resources and best practice guides • Membership of national organisations such as DIMAH
Internal collaboration	Identification and engagement of the potential resources within the wider institutional network; these include: • Institutional faculty and / or students • Expert patients • Clinicians at partner NHS organisations • Members of other university departments
Curriculum and content review	• Review of current learning materials for LGBT-inclusivity and adaption to embed additional LGBT learning outcomes into existing materials (e.g. re-writing of existing seminars and clinical cases) • Consideration of the coverage of LGBT topics as part of any formal or informal curriculum review process
Innovative content delivery	• Creativity and flexibility in the delivery of LGBT content (e.g. through e-Learning)

### Future research

The landscape of UK undergraduate medical education is currently in a state of change due to the introduction the Medical Licensing Assessment (MLA) for students graduating in the academic year 2023–24 or later in the UK, which will be based upon a common curriculum [30]. It is not yet known whether this will lead to an increased degree of curriculum homogeneity between medical schools and what impact this would have on both the current and proposed LGBT teaching; following the introduction of this assessment, a repeat of this study could be used to evaluate this in detail. In addition, a critical policy analysis perspective, such as Carol Bacchi’s approach [31], may help shed light further on the interaction between representation in policy, regulation, assessment and curriculum agendas, and the way in which specific healthcare needs of the LGBT community are perceived and met.

## Conclusions

Our study demonstrates a significant variation in the amount and breadth of content within the undergraduate curricula of UK medical schools. Overall, there is a good degree of coverage in topics that serve to address the areas identified by Stonewall as being important to LGBT patients. Despite this, medical schools are struggling to find space for this topic within an already overloaded curriculum, and study respondents reported an overall dissatisfaction with the coverage of LGBT topics at their institutions. Our study provides suggestions for undergraduate curriculum development leads about how to improve the level and range of LGBT-associated content in their courses (Table 3).

## Supplementary Information

**Additional file 1.**

**Additional file 2.**

## Data Availability

The datasets used and / or analysed during the current study are available from the corresponding author on reasonable request. We do not have ethical permission to upload the dataset into a repository. Please note that all study data has been anonymised for confidentiality purposes.

## Abbreviations

BSMS: Brighton and Sussex Medical School
DIMAH: Diversity in Medicine and Health
EDI: Equality, Diversity, and Inclusion
GMC: General Medical Council
LGBT: Lesbian, Gay, Bisexual, and Transgender
MLA: Medical Licensing Assessment
MS: Medical School
OSCE: Objective Structured Clinical Examination
RQ: Research Question
UK: United Kingdom
