# Supplementary material for: Que(e) rying undergraduate medical curricula: a cross-sectional online survey of lesbian, gay, bisexual, transgender, and queer content inclusion in UK undergraduate medical education
Source: BMC Med Educ. 2021 Feb 12;21:100. doi: 10.1186/s12909-021-02532-y (PMC7881554; doi:10.1186/s12909-021-02532-y)
Supplement: Supplementary file 1 — Additional file 1. [file 12909_2021_2532_MOESM1_ESM.doc]

**Que(e)rying undergraduate medical curricula: a cross-sectional online survey of lesbian, gay, bisexual, transgender, and queer content inclusion in UK undergraduate medical education**

STROBE Statement (***cross-sectional studies***)

|  | Item No | Recommendation | Location in manuscript |
| --- | --- | --- | --- |
| **Title and abstract** | 1 | (*a*) Indicate the study’s design with a commonly used term in the title or the abstract | Title and Abstract pages 1 and 2 |
|  |  | (*b*) Provide in the abstract an informative and balanced summary of what was done and what was found | Abstract pages 1 and 2, paragraphs 2 and 3 |
| Introduction | | |  |
| Background/rationale | 2 | Explain the scientific background and rationale for the investigation being reported | Background pages 3-6, paragraphs 1 – 7 |
| Objectives | 3 | State specific objectives, including any prespecified hypotheses | Background page 6, paragraph 8 (aims) |
| Methods | | |  |
| Study design | 4 | Present key elements of study design early in the paper | Methods, page 6 paragraph 1 (design) |
| Setting | 5 | Describe the setting, locations, and relevant dates, including periods of recruitment, exposure, follow-up, and data collection | Methods, pages 7 and 8 paragraphs 3 – 5 (under procedure) |
| Participants | 6 | (*a*) Give the eligibility criteria, and the sources and methods of selection of participants | Methods page 7, paragraph 2 (sample and eligibility) |
| Variables | 7 | Clearly define all outcomes, exposures, predictors, potential confounders, and effect modifiers. Give diagnostic criteria, if applicable | Methods pages 8 and 9, paragraphs 6 – 8 (questionnaire design) |
| Data sources/ measurement | 8* | For each variable of interest, give sources of data and details of methods of assessment (measurement). Describe comparability of assessment methods if there is more than one group | Methods, pages 8 and 9 paragraphs 6 – 8 (questionnaire design) |
| Bias | 9 | Describe any efforts to address potential sources of bias | Methods, paragraph 5 |
| Study size | 10 | Explain how the study size was arrived at | Methods, page 7 paragraph 2 (sample and eligibility) |
| Quantitative variables | 11 | Explain how quantitative variables were handled in the analyses. If applicable, describe which groupings were chosen and why | Methods, pages 9 and 10 paragraph 9 (data analysis) |
| Statistical methods | 12 | (*a*) Describe all statistical methods, including those used to control for confounding | Methods, pages 9 and 10 paragraph 9 (data analysis) |
|  |  | (*b*) Describe any methods used to examine subgroups and interactions | N/A |
|  |  | (*c*) Explain how missing data were addressed | Methods, , pages 9 and 10 paragraph 9 (data analysis) |
|  |  | (*d*) If applicable, describe analytical methods taking account of sampling strategy | N/A |
|  |  | (*e*) Describe any sensitivity analyses | N/A |
| Results | | |  |
| Participants | 13* | (a) Report numbers of individuals at each stage of study—eg numbers potentially eligible, examined for eligibility, confirmed eligible, included in the study, completing follow-up, and analysed | Results, page 10 paragraph 1 |
|  |  | (b) Give reasons for non-participation at each stage | Results, page 10 paragraph 1 |
|  |  | (c) Consider use of a flow diagram | N/A |
| Descriptive data | 14* | (a) Give characteristics of study participants (eg demographic, clinical, social) and information on exposures and potential confounders | Results, page 10 paragraph 1 |
|  |  | (b) Indicate number of participants with missing data for each variable of interest | Results, page 10 paragraph 1 |
| Outcome data | 15* | Report numbers of outcome events or summary measures | Results, pages 11-16 paragraphs 2 – 18 |
| Main results | 16 | (*a*) Give unadjusted estimates and, if applicable, confounder-adjusted estimates and their precision (eg, 95% confidence interval). Make clear which confounders were adjusted for and why they were included | Results, pages 11-16 paragraphs 2 – 18 |
|  |  | (*b*) Report category boundaries when continuous variables were categorized | N/A |
|  |  | (*c*) If relevant, consider translating estimates of relative risk into absolute risk for a meaningful time period | N/A |
| Other analyses | 17 | Report other analyses done—eg analyses of subgroups and interactions, and sensitivity analyses | Results, pages 11-16 paragraphs 2 – 18 |
| Discussion | | |  |
| Key results | 18 | Summarise key results with reference to study objectives | Discussion, pages 17-19 paragraphs 2 – 7 (principal findings) |
| Limitations | 19 | Discuss limitations of the study, taking into account sources of potential bias or imprecision. Discuss both direction and magnitude of any potential bias | Discussion, , pages 19-21 paragraphs 8 and 9 (strengths and limitations) |
| Interpretation | 20 | Give a cautious overall interpretation of results considering objectives, limitations, multiplicity of analyses, results from similar studies, and other relevant evidence | Discussion paragraphs pages 21-22 |
| Generalisability | 21 | Discuss the generalisability (external validity) of the study results | Discussion, pages 21-22 paragraphs 10 and 11 (study implications and recommendations) |
| Other information | | |  |
| Funding | 22 | Give the source of funding and the role of the funders for the present study and, if applicable, for the original study on which the present article is based | Declarations pages 25 and 26, paragraph 5 (funding) |

*Give information separately for exposed and unexposed groups.
