# Supplementary material for: Que(e) rying undergraduate medical curricula: a cross-sectional online survey of lesbian, gay, bisexual, transgender, and queer content inclusion in UK undergraduate medical education
Source: BMC Med Educ. 2021 Feb 12;21:100. doi: 10.1186/s12909-021-02532-y (PMC7881554; doi:10.1186/s12909-021-02532-y)
Supplement: Supplementary file 2 — Additional file 2. [file 12909_2021_2532_MOESM2_ESM.docx]

UK Medical School National LGBT Survey

Thank you very much for your interest in our research project investigating the current state of LGBT-focussed teaching within UK Medical Schools.

**Instructions [for primary participant]**

This survey will ask you questions about the curriculum at your medical school with a focus on how your institution teaches medical students about the health inequalities faced by Lesbian, Gay, Bisexual, and Transgender (LGBT) individuals.

This survey is **comprised of 3 parts** that cover the current LGBT-related teaching within the curriculum (Part 1), any proposed or planned LGBT-related teaching within the curriculum (Part 2), and your opinions on LGBT-related teaching within the curriculum (Part 3). The survey will take between 10 and 20 minutes to complete and contains 30 questions. You may pause and restart the survey at any time. All responses will be anonymised.

This survey is meant to be completed by the Lead for Undergraduate Medical Education at your institution or another individual with significant responsibility for curriculum development of the undergraduate medical course; if you do not feel that you are the correct faculty member to be answering this, please forward the link to this survey on to an individual whom you believe may be more appropriate.

Optional: At the end of the survey, you will have the opportunity to nominate one colleague to review, edit, and add to your responses for Parts 1 and 2. Your responses to Part 3 of this survey will **not** be visible to your nominated colleague.

Thank you very much in advance; should you have any further questions regarding this survey or project, do feel free to contact us using the following details:

- Investigator – Independent Research Project (IRP) Student: **Nicholas Tollemache** ([N.Tollemache1@uni.bsms.ac.uk](mailto:N.Tollemache1@uni.bsms.ac.uk))
- Supervisor: **Carrie Llewellyn** ([C.D.Llewellyn@bsms.ac.uk](mailto:C.D.Llewellyn@bsms.ac.uk))
- Supervisor: **Duncan Shrewsbury** ([D.Shrewsbury@bsms.ac.uk](mailto:D.Shrewsbury@bsms.ac.uk))

**Instructions [for nominated / secondary participant]**

This survey will ask you questions about the curriculum at your medical school with a focus on how your institution teaches medical students about the health inequalities faced by Lesbian, Gay, Bisexual, and Transgender (LGBT) individuals.

This survey is **comprised of 3 parts** that cover the current LGBT-related teaching within the curriculum (Part 1), any proposed or planned LGBT-related teaching within the curriculum (Part 2), and your opinions on LGBT-related teaching within the curriculum (Part 3).

Your colleague has asked for assistance in reviewing their responses to **Parts 1 and 2** of this survey; you may provide your own responses to Part 3 of the survey (these will not be visible to your colleague or anyone else at your institution). The survey will take between 10 and 20 minutes to complete and contains 30 questions. You may pause and restart the survey at any time. All responses will be anonymised.

Thank you very much in advance; should you have any further questions regarding this survey or project, do feel free to contact us using the following details:

- Investigator – Independent Research Project (IRP) Student: **Nicholas Tollemache** ([N.Tollemache1@uni.bsms.ac.uk](mailto:N.Tollemache1@uni.bsms.ac.uk))
- Supervisor: **Carrie Llewellyn** ([C.D.Llewellyn@bsms.ac.uk](mailto:C.D.Llewellyn@bsms.ac.uk))
- Supervisor: **Duncan Shrewsbury** ([D.Shrewsbury@bsms.ac.uk](mailto:D.Shrewsbury@bsms.ac.uk))

**Informed Consent**

The participant information sheet, detailing your rights as a research participant and our responsibilities to you as researchers, is available here: [UoB Microsoft OneDrive Link to File]

Please study the document carefully and, should you require any further information before deciding whether to proceed, please do not hesitate to contact us using the following details:

- Investigator – Independent Research Project (IRP) Student: **Nicholas Tollemache** ([N.Tollemache1@uni.bsms.ac.uk](mailto:N.Tollemache1@uni.bsms.ac.uk))
- Supervisor: **Carrie Llewellyn** ([C.D.Llewellyn@bsms.ac.uk](mailto:C.D.Llewellyn@bsms.ac.uk))
- Supervisor: **Duncan Shrewsbury** ([D.Shrewsbury@bsms.ac.uk](mailto:D.Shrewsbury@bsms.ac.uk))

**Please note that:**

- We will **not** publicly report questionnaire responses from individual institutions or geographical regions
- Measures will be taken to ensure that **individual** **medical schools will not be identifiable in any reporting of results** – questionnaire responses from each medical school will be assigned a unique code; an index of these codes will be held separately and securely to prevent identification of responses
- Your **name, job title, and email address (if provided) will be held separately** from your questionnaire responses

Consent Form

- Please select your current institution

[dropdown box containing a list of all 42 UK Medical Schools]

- Please enter your full name

[textbox for user input]

- Please enter the job title / role you hold at your institution

[textbox for user input]

- Please tick the following checkboxes to confirm that you accept the statements below:

☐ I confirm that I have read and understood the information provided in the participant information sheet

☐ I have had the chance to read the information, ask questions about the study, and am satisfied with the answers I have been given

☐ I understand that my participation in this study is voluntary and that I am free to stop at any time, and I do not have to give a reason for doing so

☐ I agree to take part in the study

**START SURVEY**

**Part 1 - Current Teaching**

*This section relates to all LGBT teaching that is currently taking place at your institution (i.e. at least one teaching session has already occurred)*

1. Is LGBT-specific content taught in **years 1 and 2** of the undergraduate medical course?

⃝ Yes

⃝ No [[skip to question 5](#Q5)]

⃝ Don’t know [[skip to question 5](#Q5)]

*new screen*

Considering **years 1 and 2** of the undergraduate medical course, please answer the following questions…

1. In what format(s) does LGBT-specific teaching take place? (please select all that apply)

☐ Didactic (lecture-based) teaching

☐ Small-group teaching

☐ Case-based discussions

☐ Simulation-based teaching

☐ Patient educator sessions

☐ Multi / inter-professional learning

☐ Flipped classroom sessions

☐ Bedside / clinical teaching sessions

☐ Online resources (e.g. tutorials, cases, videos)

☐ Other [free textbox for user input]

1. When does LGBT-specific teaching take place? (please select all that apply)

☐ In a distinct module

☐ Integrated throughout the curriculum

☐ Other [free textbox for user input]

1. For each of the following content areas, please indicate whether they are included in the mandatory curriculum (embedded and sustained), the elective curriculum (e.g. student-selected components / modules), or not included in the curriculum…

*Please note that these topics are illustrative; there is space at the bottom of the table for you to insert up to three additional content areas, not already included here, that you may consider to be covered by your curriculum*

|  | Mandatory curriculum | Elective curriculum | Not included in curriculum | Don’t know |
| --- | --- | --- | --- | --- |
| Awareness of LGBT-specific health inequalities | ⃝ | ⃝ | ⃝ | ⃝ |
| Understanding of LGBT Families | ⃝ | ⃝ | ⃝ | ⃝ |
| Sexual orientation | ⃝ | ⃝ | ⃝ | ⃝ |
| Gender Identity | ⃝ | ⃝ | ⃝ | ⃝ |
| Transitioning and Sex Reassignment Surgery | ⃝ | ⃝ | ⃝ | ⃝ |
| LGBT Mental Health | ⃝ | ⃝ | ⃝ | ⃝ |
| Alcohol, tobacco, and illicit drug use in LGBT people | ⃝ | ⃝ | ⃝ | ⃝ |
| Sexually transmitted infections (not HIV) in LGBT people | ⃝ | ⃝ | ⃝ | ⃝ |
| HIV in LGBT people | ⃝ | ⃝ | ⃝ | ⃝ |
| LGBT Adolescent Health | ⃝ | ⃝ | ⃝ | ⃝ |
| Chronic Disease in LGBT populations | ⃝ | ⃝ | ⃝ | ⃝ |
| Maternity and childbirth in LGBT people | ⃝ | ⃝ | ⃝ | ⃝ |
| Preventative health and cancer screening in LGBT people | ⃝ | ⃝ | ⃝ | ⃝ |
| LGBT discrimination in healthcare | ⃝ | ⃝ | ⃝ | ⃝ |
| Communication skills with LGBT people | ⃝ | ⃝ | ⃝ | ⃝ |
| [free textbox for user input] | ⃝ | ⃝ | ⃝ | ⃝ |
| [free textbox for user input] | ⃝ | ⃝ | ⃝ | ⃝ |
| [free textbox for user input] | ⃝ | ⃝ | ⃝ | ⃝ |

*new screen*

1. Is LGBT-specific content taught in **years 3 – 5** of the undergraduate medical course?

⃝ Yes

⃝ No [[skip to question 9](#Q6)]

⃝ Don’t know [[skip to question 9](#Q9)]

*new screen*

Considering **years 3 – 5** of the undergraduate medical course, please answer the following questions…

1. In what format(s) does LGBT-specific teaching take place? (please select all that apply)

☐ Didactic (lecture-based) teaching

☐ Small-group teaching

☐ Case-based discussions

☐ Simulation-based teaching

☐ Patient educator sessions

☐ Multi / inter-professional learning sessions

☐ Flipped classroom sessions

☐ Bedside / clinical teaching sessions

☐ Online resources (e.g. tutorials, cases, videos)

☐ Other [free textbox for user input]

1. When does LGBT-specific teaching take place? (please select all that apply)

☐ In distinct modules

☐ Integrated throughout the curriculum

☐ Other [free textbox for user input]

1. For each of the following content areas, please indicate whether they are included in the mandatory curriculum (embedded and sustained), the elective curriculum (e.g. student-selected components / modules, optional clinical placements), or not included in the curriculum:

*Please note that these topics are illustrative; there is space at the bottom of the table for you to insert up to three additional content areas, not already included here, that you may consider to be covered by your curriculum*

|  | Mandatory curriculum | Elective curriculum | Not included in curriculum | Don’t know |
| --- | --- | --- | --- | --- |
| Awareness of LGBT-specific health inequalities | ⃝ | ⃝ | ⃝ | ⃝ |
| Understanding of LGBT Families | ⃝ | ⃝ | ⃝ | ⃝ |
| Sexual orientation | ⃝ | ⃝ | ⃝ | ⃝ |
| Gender Identity | ⃝ | ⃝ | ⃝ | ⃝ |
| Transitioning and Sex Reassignment Surgery | ⃝ | ⃝ | ⃝ | ⃝ |
| LGBT Mental Health | ⃝ | ⃝ | ⃝ | ⃝ |
| Alcohol, tobacco, and illicit drug use in LGBT people | ⃝ | ⃝ | ⃝ | ⃝ |
| Sexually transmitted infections (not HIV) in LGBT people | ⃝ | ⃝ | ⃝ | ⃝ |
| HIV in LGBT people | ⃝ | ⃝ | ⃝ | ⃝ |
| LGBT Adolescent Health | ⃝ | ⃝ | ⃝ | ⃝ |
| Chronic Disease in LGBT populations | ⃝ | ⃝ | ⃝ | ⃝ |
| Maternity and childbirth in LGBT people | ⃝ | ⃝ | ⃝ | ⃝ |
| Preventative health and cancer screening in LGBT people | ⃝ | ⃝ | ⃝ | ⃝ |
| LGBT discrimination in healthcare | ⃝ | ⃝ | ⃝ | ⃝ |
| Communication skills with LGBT people | ⃝ | ⃝ | ⃝ | ⃝ |
| [free textbox for user input] | ⃝ | ⃝ | ⃝ | ⃝ |
| [free textbox for user input] | ⃝ | ⃝ | ⃝ | ⃝ |
| [free textbox for user input] | ⃝ | ⃝ | ⃝ | ⃝ |

*new screen*

1. If possible, please provide further details about the nature of any specific LGBT teaching sessions or programmes that run at any point in the programme (years 1 – 5) [question only appears if answered ‘Yes’ to Q1 and / or Q5]

[free textbox for user input]

*new screen*

1. Does your institution formally evaluate and / or assess the efficacy of LGBT-specific teaching?

⃝ Yes

⃝ No [[skip to question 14](#Q14)]

⃝ Don’t know [[skip to question 14](#Q14)]

*new screen*

Considering the **evaluation and assessment** of LGBT-specific teaching…

1. Does your institution collect feedback from students about the LGBT teaching they have received?

⃝ Yes

⃝ No

⃝ Don’t know

1. Does your institution formally assess students against the learning objectives from the LGBT teaching they have received?

⃝ Yes – formative assessment

⃝ Yes – summative assessment

⃝ Yes – both formative and summative assessment

⃝ No [[skip to question 14](#Q14)]

⃝ Don’t know [[skip to question 14](#Q14)]

*new screen*

1. What methods of assessment are used at your institution to formally assess students against the LGBT learning objectives? (please tick all that apply)

☐ Written examination – Multiple Choice Questions (MCQs)

☐ Written examination – Extended Matching Items / Questions

☐ Written examination – Short Answer Questions (SAQs)

☐ Written examination – Long Answer Questions (LAQs)

☐ Coursework (e.g. essay, small group presentation, etc.)

☐ Objective Structured Clinical Examinations (OSCEs)

☐ Oral Case-Based Discussions (CBDs)

☐ Logbooks

☐ (e)Portfolio

☐ Other [free textbox for user input]

*new screen*

Considering the **resources and support available** for LGBT-specific teaching…

1. Does your institution provide **faculty development** for educators about LGBT-inclusive teaching?

⃝ Yes (please provide additional details if possible) [free textbox for additional details]

⃝ No

⃝ Don’t know

1. Does your institution offer **written resources** for educators about LGBT-inclusive teaching?

*For example, this could include guidance on the use of LGBT-inclusive language, specific terminology, images used in teaching materials, and other examples of inclusive practice*

⃝ Yes (please provide additional details if possible) [free textbox for additional details]

⃝ No

⃝ Don’t know

1. Does your institution **audit teaching sessions and / or student resources** for LGBT-inclusivity?

⃝ Yes (please provide additional details if possible) [free textbox for additional details]

⃝ No

⃝ Don’t know

1. Do you require educators to submit content (e.g. lecture materials) to faculty members or the school office **in advance** of teaching sessions?

⃝ Yes (please provide additional details if possible) [free textbox for additional details]

⃝ No [skip to question 19]

*new screen*

1. Is the content provided in advance by educators reviewed for LGBT-inclusivity before the teaching session?

⃝ Yes (please provide additional details if possible) [free textbox for additional details]

⃝ No

*new screen*

1. What factors have you, or your team, encountered when implementing the current LGBT teaching sessions or programmes? (please select all that apply) [question only appears if answered ‘Yes’ to Q1 and / or Q5]

☐ Insufficient funding available

☐ Lack of faculty buy-in

☐ Lack of relevant experience within the faculty

☐ Lack of perceived relevance to undergraduate medical students

☐ Lack of space within the curriculum / time constraints

☐ Insufficient knowledge about the implementation of LGBT-inclusive teaching

☐ Lack of perceived importance

☐ Lack of engagement from partner institutions (e.g. NHS Trusts, GP Practices)

☐ Other [free textbox for user input]

1. For any of the factors that you have selected above, please describe how these were overcome in order to successfully implement the teaching [question only appears if at least one option selected in Q19]

[free textbox for user input]

*new screen*

1. Please estimate how many **total hours** are dedicated to the teaching of LGBT content across the whole of the undergraduate medical course?

[textbox accepting only integers between 0 and 500; able to leave the box blank]

**Part 2 - Proposed Teaching**

*This section relates to teaching that is yet to take place (i.e. where the first teaching session has not yet been delivered to students)*

1. Is your institution planning to implement any **new** LGBT-specific teaching within the curriculum in the next three (3) academic years?

⃝ Yes

⃝ Possibly

⃝ No [[skip to question 27](#Q27)]

⃝ Don’t know [[skip to question 27](#Q27)]

*new screen*

1. Please identify any content areas that you are planning on implementing teaching for and indicate the stage that you are currently at in this process

*Please note that these topics are illustrative; there is space at the bottom of the table for you to insert up to three additional content areas, not already included here, that you are planning to cover in your curriculum*

|  | Don’t know | No current plans | Initial concept, no action taken yet | Initial scoping / information gathering | Content creation | Ready to go |
| --- | --- | --- | --- | --- | --- | --- |
| Awareness of LGBT-specific health inequalities | ⃝ | ⃝ | ⃝ | ⃝ | ⃝ | ⃝ |
| Understanding of LGBT Families | ⃝ | ⃝ | ⃝ | ⃝ | ⃝ | ⃝ |
| Sexual orientation | ⃝ | ⃝ | ⃝ | ⃝ | ⃝ | ⃝ |
| Gender Identity | ⃝ | ⃝ | ⃝ | ⃝ | ⃝ | ⃝ |
| Transitioning and Sex Reassignment Surgery | ⃝ | ⃝ | ⃝ | ⃝ | ⃝ | ⃝ |
| LGBT Mental Health | ⃝ | ⃝ | ⃝ | ⃝ | ⃝ | ⃝ |
| Alcohol, tobacco, and illicit drug use in LGBT people | ⃝ | ⃝ | ⃝ | ⃝ | ⃝ | ⃝ |
| Sexually transmitted infections (not HIV) in LGBT people | ⃝ | ⃝ | ⃝ | ⃝ | ⃝ | ⃝ |
| HIV in LGBT people | ⃝ | ⃝ | ⃝ | ⃝ | ⃝ | ⃝ |
| LGBT Adolescent Health | ⃝ | ⃝ | ⃝ | ⃝ | ⃝ | ⃝ |
| Chronic Disease in LGBT populations | ⃝ | ⃝ | ⃝ | ⃝ | ⃝ | ⃝ |
| Maternity and childbirth in LGBT people | ⃝ | ⃝ | ⃝ | ⃝ | ⃝ | ⃝ |
| Preventative health and cancer screening in LGBT people | ⃝ | ⃝ | ⃝ | ⃝ | ⃝ | ⃝ |
| LGBT discrimination in healthcare | ⃝ | ⃝ | ⃝ | ⃝ | ⃝ | ⃝ |
| Communication skills with LGBT people | ⃝ | ⃝ | ⃝ | ⃝ | ⃝ | ⃝ |
| [free textbox for user input] | ⃝ | ⃝ | ⃝ | ⃝ | ⃝ | ⃝ |
| [free textbox for user input] | ⃝ | ⃝ | ⃝ | ⃝ | ⃝ | ⃝ |
| [free textbox for user input] | ⃝ | ⃝ | ⃝ | ⃝ | ⃝ | ⃝ |

1. What factor(s) have you or your team already encountered when planning the implementation of new LGBT teaching? (please select all that apply)

☐ Insufficient funding available

☐ Lack of faculty buy-in

☐ Lack of relevant experience within the faculty

☐ Lack of perceived relevance to undergraduate medical students

☐ Lack of space within the curriculum / time constraints

☐ Insufficient knowledge about the implementation of LGBT-inclusive teaching

☐ Lack of perceived importance

☐ Lack of engagement from partner institutions (e.g. NHS Trusts, GP Practices)

☐ Other [free textbox for user input]

1. Did any of the factor(s) identified in the previous question prevent the proposed teaching from proceeding?

⃝ Yes (please provide additional details if possible) [free textbox for additional details]

⃝ No

⃝ Don’t know

1. What factor(s) do you predict that you will encounter when implementing new LGBT teaching? (please select all that apply)

☐ Insufficient funding available

☐ Lack of faculty buy-in

☐ Lack of relevant experience within the faculty

☐ Lack of perceived relevance to undergraduate medical students

☐ Lack of space within the curriculum / time constraints

☐ Insufficient knowledge about the implementation of LGBT-inclusive teaching

☐ Lack of perceived importance

☐ Lack of engagement from partner institutions (e.g. NHS Trusts, GP Practices)

☐ Other [free textbox for user input]

*new screen*

1. Overall, how easy has it been to implement new LGBT teaching programmes? [question only appears if answered ‘Yes’ to Q1 and / or Q5 and / or Q22]

⃝ Very easy

⃝ Easy

⃝ Neither easy, nor difficult

⃝ Difficult

⃝ Very difficult

**Part 3 – Opinions on LGBT teaching within the curriculum**

*This section relates to your personal opinions on LGBT teaching within the undergraduate medical curriculum*

1. What is your overall opinion of the coverage of LGBT topics at your institution?

⃝ Very good

⃝ Good

⃝ Fair

⃝ Could do better

⃝ Could do a lot better

⃝ Don’t Know

1. To what degree do you consider LGBT inequalities to be covered by existing wider university inclusivity guidance and / or policies?

⃝ Completely covered

⃝ Possibly covered

⃝ Somewhat covered

⃝ Not at all covered

⃝ Don’t Know

1. Please rate **your opinion** of how well each content area is covered at your institution:

*Please note that these topics are illustrative; there is space at the bottom of the table for you to insert up to three additional content areas, not already included here, that you may consider to be covered, or should be covered, by your curriculum*

|  | Coverage not required | Too little coverage | Adequate coverage | In-depth coverage | Too much coverage | Don’t know |
| --- | --- | --- | --- | --- | --- | --- |
| Awareness of LGBT-specific health inequalities | ⃝ | ⃝ | ⃝ | ⃝ | ⃝ | ⃝ |
| Understanding of LGBT Families | ⃝ | ⃝ | ⃝ | ⃝ | ⃝ | ⃝ |
| Sexual orientation | ⃝ | ⃝ | ⃝ | ⃝ | ⃝ | ⃝ |
| Gender Identity | ⃝ | ⃝ | ⃝ | ⃝ | ⃝ | ⃝ |
| Transitioning and Sex Reassignment Surgery | ⃝ | ⃝ | ⃝ | ⃝ | ⃝ | ⃝ |
| LGBT Mental Health | ⃝ | ⃝ | ⃝ | ⃝ | ⃝ | ⃝ |
| Alcohol, tobacco, and illicit drug use in LGBT people | ⃝ | ⃝ | ⃝ | ⃝ | ⃝ | ⃝ |
| Sexually transmitted infections (not HIV) in LGBT people | ⃝ | ⃝ | ⃝ | ⃝ | ⃝ | ⃝ |
| HIV in LGBT people | ⃝ | ⃝ | ⃝ | ⃝ | ⃝ | ⃝ |
| LGBT Adolescent Health | ⃝ | ⃝ | ⃝ | ⃝ | ⃝ | ⃝ |
| Chronic Disease in LGBT populations | ⃝ | ⃝ | ⃝ | ⃝ | ⃝ | ⃝ |
| Maternity and childbirth in LGBT people | ⃝ | ⃝ | ⃝ | ⃝ | ⃝ | ⃝ |
| Preventative health and cancer screening in LGBT people | ⃝ | ⃝ | ⃝ | ⃝ | ⃝ | ⃝ |
| LGBT discrimination in healthcare | ⃝ | ⃝ | ⃝ | ⃝ | ⃝ | ⃝ |
| Communication skills with LGBT people | ⃝ | ⃝ | ⃝ | ⃝ | ⃝ | ⃝ |
| [free textbox for user input] | ⃝ | ⃝ | ⃝ | ⃝ | ⃝ | ⃝ |
| [free textbox for user input] | ⃝ | ⃝ | ⃝ | ⃝ | ⃝ | ⃝ |
| [free textbox for user input] | ⃝ | ⃝ | ⃝ | ⃝ | ⃝ | ⃝ |

**Supplementary information [only visible for primary participant]**

*In order for us to gain a complete understanding of the current and proposed LGBT teaching within the curriculum at your institution, you may feel that it is useful to refer this survey to a colleague.*

- Is there another individual at your institution who is responsible for creating, implementing, and evaluating LGBT teaching within your curriculum, and who would be happy to add to your answers to **Parts 1 (Current Teaching)** and **2 (Proposed Teaching)** of this survey?

⃝ Yes

⃝ No [[end questionnaire screen](#end)]

⃝ Don’t know

*new screen*

After completing this survey, **you** will receive an email from us containing a link that you may forward onto a colleague at your institution.

Please note that this individual will **only** **be able to** **view and edit your responses to Parts 1 (Current Teaching) and 2 (Proposed Teaching) of this survey**; they will complete **their own** responses to Part 3 (Opinions on LGBT teaching within the curriculum).

**End Questionnaire Screen**

Thank you for your participation in this study!

- Would you like to receive a copy of the results of this study when complete?

⃝ Yes

⃝ No

- Please enter your email address [appears if answered ‘Yes’ to the above question]

[text box with email address validation]

*In accordance with European Union General Data Protection Regulations (GDPR), your email address will only be used in order to contact you with a copy of the results of this study. Your email address will be stored on file for a maximum of two years OR until we have distributed a final copy of the results of this study (the shorter of the two).*

**SUBMIT AND END SURVEY**
